# Convolutional Neural Networks-Based Object Detection Algorithm by Jointing Semantic Segmentation for Images

**DOI:** 10.3390/s20185080

**Published:** 2020-09-07

**Authors:** Baohua Qiang, Ruidong Chen, Mingliang Zhou, Yuanchao Pang, Yijie Zhai, Minghao Yang

**Affiliations:** 1 Guangxi Colleges and Universities Key Laboratory of Intelligent Processing of Computer Image and Graphics, Guilin University of Electronic Technology, Guilin 541004, China; qiangbh@guet.edu.cn (B.Q.); pgezcrb@163.com (R.C.); pangyc1011@163.com (Y.P.); 18737309531@163.com (Y.Z.); mhyang@nlpr.ia.ac.cn (M.Y.); 2 School of Computer Science, Chongqing University, 174 Shazheng Street, Shapingba District, Chongqing 400044, China; 3 State Key Laboratory of Internet of Things for Smart City, Faculty of Science and Technology, University of Macau, Macau, China

**Keywords:** object detection, semantic segmentation, attention mechanism, hourglass network, sensor

## Abstract

In recent years, increasing image data comes from various sensors, and object detection plays a vital role in image understanding. For object detection in complex scenes, more detailed information in the image should be obtained to improve the accuracy of detection task. In this paper, we propose an object detection algorithm by jointing semantic segmentation (SSOD) for images. First, we construct a feature extraction network that integrates the hourglass structure network with the attention mechanism layer to extract and fuse multi-scale features to generate high-level features with rich semantic information. Second, the semantic segmentation task is used as an auxiliary task to allow the algorithm to perform multi-task learning. Finally, multi-scale features are used to predict the location and category of the object. The experimental results show that our algorithm substantially enhances object detection performance and consistently outperforms other three comparison algorithms, and the detection speed can reach real-time, which can be used for real-time detection.

## 1. Introduction

With the development of the Internet of Things [1], more and more image data are collected by various image sensors or video sensors. Before using image data for more complex computer vision tasks, we need to know what objects are in the image and where they are located. Therefore, object detection [2] has always been a hot research direction in the field of computer vision, and its purpose is to locate and classify objects in images or videos. Object detection has been widely used in many fields, including intelligent traffic [3] and human pose estimation [4].

Traditional algorithms solve the detection problem for images by finding foreground and background from the picture and then manually extracting foreground features for classification. The algorithm of extracting the foreground can be divided into static and dynamic according to the state of the object. The static object detection algorithm for images usually uses the background subtraction algorithm [5,6]. The foreground is the part where the pixel value varies greatly. The dynamic object detection algorithm usually adopts the frame difference [7,8] algorithm to subtract two adjacent frames. The area where the pixel value changes drastically is the area where the object appears.

The feature extraction algorithm combined with the classifier is used by the traditional object detection algorithm to classify the detected foreground. Viola and Jones [9] proposed the Haar feature combined with Adaboost object detection algorithm of human faces. Dalal et al. [10] proposed the human detection model of histograms of oriented gradients (HOG) combined with support vector machines (SVM), which had high detection accuracy but long detection time. Felzenszwalb et al. [2] proposed deformable parts model (DPM) with HOG feature modified, which greatly improved algorithm performance.

In recent years, many algorithms have been proposed to address the problem of object detection. The object detection algorithms based on deep learning can be divided into two-stage detection algorithms and one-stage detection algorithms. The two-stage algorithm is to first generate a region proposal [11], and then target the boundary box and category prediction of the region proposal. Girshick et al. [7] proposed the classic regions with convolutional neural networks (CNN) features (R-CNN) to achieve excellent object detection accuracy by using a deep ConvNet to classify object proposals, but it is very time-consuming. To solve this problem, Girshick et al. [12,13] proposed the upgraded version of R-CNN, Faster R-CNN, which innovatively used the region proposal network (RPN) to directly classify the region proposal in the convolutional neural network, and achieved the end-to-end goal of the whole detection framework. He et al. [14] proposed Mask R-CNN on the basis of Faster R-CNN, which added a branch for semantic segmentation tasks, and used detection tasks and segmentation tasks to extract image features to improve the accuracy of detection. He et al. [15] proposed spatial pyramid pooling networks (SPPNet) to generate fixed-length representations. Kong et al. [16] proposed HyperNet, which combines the generation of candidate regions with the detection task to produce fewer candidate regions while ensuring a higher recall rate. Cai and Vasconcelos [17] proposed Cascade R-CNN to address the problem of overfitting and quality mismatch.

The one-stage detection algorithms do not need to select region proposals, but use the regression to directly calculate the positioning box and object category, which further reduce the running time. Redmon et al. [18] proposed the you only look once (YOLO) algorithm to meet the requirements of real-time detection, but the detection accuracy of small objects is not high. Liu et al. [19] proposed the single shot multibox detector (SSD) algorithm to predict the object from multiple feature maps, which largely solved the problem of small object detection. Lin et al. [20] proposed RetinaNet mainly to solve the extremely imbalanced problem of one-stage algorithm positive and negative samples and difficult and easy samples. Zhang et al. [21] proposed the RefineDet method, which absorbed the advantages of the two-stage algorithm, so that the one-stage detection algorithm can also have the accuracy of the two-stage algorithm. Liu et al. [22] proposed RFBNet to use cavity convolution to improve the receptive field. Shen et al. [23] proposed deeply supervised object detector (DSOD) to restart training neural networks for detection tasks, and also introduced the idea of DenseNet [24], which greatly reduced the number of parameters. Law and Deng [25] proposed Cornernet to detect an object bounding box as a pair of keypoints using a single convolution neural network. To further improve on Cornernet, Duan et al. [26] proposed CenterNet to detect each object as a triplet of keypoints. Tian et al. [27] proposed fully convolutional one-stage object detector (FCOS) to solve object detection in a per-pixel prediction fashion.

Although the above methods focus on object detection, those methods still have the following shortcomings:

- Object detection not only needs to maintain a high detection accuracy, but also has certain requirements for detection speed. However, most of the existing algorithms have slow detection speeds and cannot keep up with the playback speed of the video stream, which may cause the detection of important frames to be missed.
- Overlap and occlusions in the image may cause false detection.
- Objects often appear in complex backgrounds, which will cause interference and make detection difficult.

To address the above problems, we propose an object detection algorithm combined with semantic segmentation [28,29]. The contributions of this study are as follows:

- We inherit the idea of one-stage target detection algorithm, and the entire model adopts the network structure in the form of full convolution to ensure the detection speed is fast enough.
- We use the hourglass structure to generate multi-scale features with high-level semantic information, which enables the algorithm to detect small-scale objects more effectively, and we use semantic segmentation as an auxiliary task for object detection to extract more detailed features with pixel-level semantic segmentation to better detect partially occluded objects.
- We use the attention mechanism to make the model pay more attention to the object, reduce the influence of the image background without causing model degradation.
- The experimental results show that our algorithm substantially enhances object detection performance and consistently outperforms other comparison algorithms, and the detection speed can reach real-time, which can be used for real-time detection.

The remainder of this paper is organized as follows. The proposed algorithm is illustrated in Section 2. The experiment results are presented in Section 3. Finally, Section 4 draws conclusions.

## 2. Materials and Methods

### 2.1. Framework

**Figure 1:**
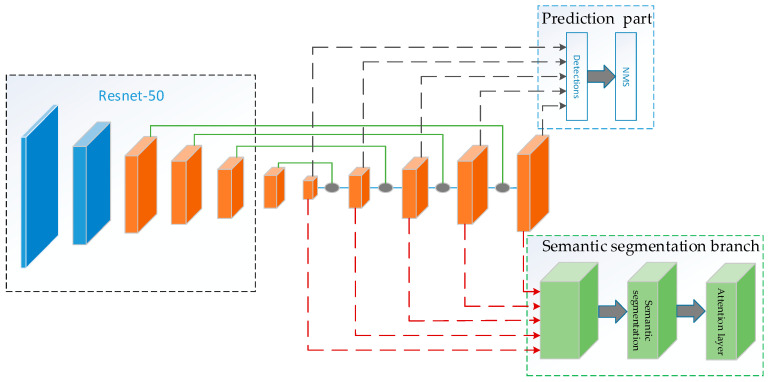
The flow chart of the proposed algorithm.

As shown in Figure 1, our algorithm consists of three parts. First, we use an hourglass structure to generate feature mapping for the preliminary feature generated by ResNet-50 [30] to generate advanced features with rich semantic information. Second, we use the semantic segmentation task as an auxiliary task to make the model multi-task learning. Finally, we use the multi-scale features generated by the hourglass structure to predict the location and category of the image object.

### 2.2. Feature Extraction

The images undergo preliminary feature extraction through ResNet-50, and the generated feature map passes through an hourglass structure. The subsampled structure of the hourglass structure is used to extract multi-scale features, and the up-sampling structure is used to integrate multi-scale features to generate advanced features with rich semantic information. The fully convolutional network structure is used for the whole model to ensure that the detection speed is fast enough. The hourglass structure contains the last three residual blocks of ResNet-50. In order to reduce the model training time, pre-training parameters of ResNet-50 are loaded in this paper for training, so the structure of ResNet-50 is not changed.

**Figure 2:**
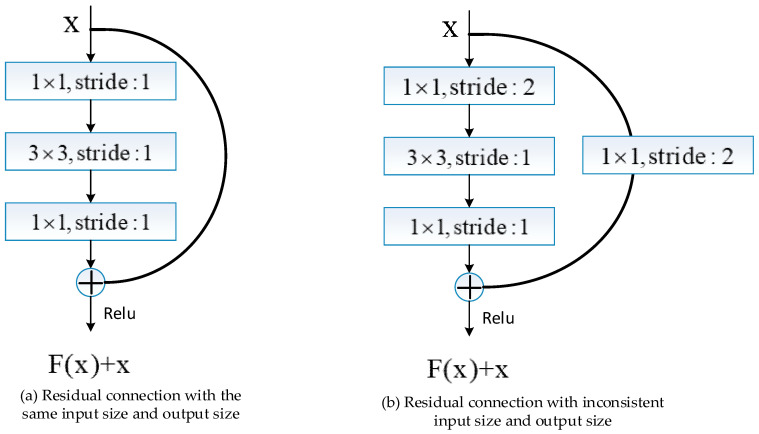
Schematic diagram of subsampled block structure in the hourglass structure.

In order to facilitate the training of the model, each block of the hourglass structure uses residual connections. In the up-sampling part of the hourglass structure, the input size and output size in the intra-box are the same, and the residual structure shown in Figure 2a is used. When the input size and output size are the same in the inter-box, the residual structure shown in Figure 2a is used; otherwise, the residual structure shown in Figure 2b is used. It achieves the effect of subsampled by setting the sliding step of convolution in the convolutional layer as 2, and at the same time, image feature extraction is completed.

**Figure 3:**
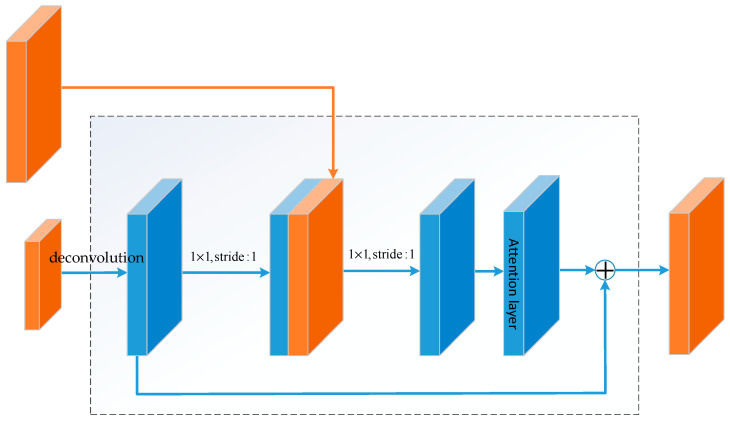
Schematic diagram of up-sampling structure in hourglass structure incorporating attention mechanism.

Each block in the up-sampling structure of the hourglass structure has a connection structure in the middle. The detailed construction is shown in Figure 3.

The advantage of using feature map stacking in up-sampling is combined with the high-level and low-level features, so that the generated feature map can have high-level semantic information even when the size of the generated feature map is large. The connection structure is also a residual structure, and the shortcut connections make the network easier to train. The structure has two advantages. First, because of the identity mapping, the attention mechanism [31,32] layer of each up-sampling block is not simply superimposed, which will not lead to the reduction of internal features. Second, because of the nature of the residual structure itself, learning the weights in the attention mechanism layer will not bring additional difficulties to model training and will not cause model degradation. The detailed parameters of the hourglass structure behind the ResNet-50 structure are shown in Table 1.

### 2.3. Semantic Segmentation Branch

The multi-scale features generated by the hourglass structure are input into the semantic segmentation branch, and the segmentation mask is predicted by fusing the features of each scale to complete the semantic segmentation task.

Feature maps of different sizes are first subjected to a convolution of size 1×1, then the nearest neighbor interpolation method is used to enlarge the feature map to the size of the last layer. Finally, the feature maps are generated through convolution kernels to predict the segmentation mask.

In the last part of the semantic segmentation branch, an attention mechanism layer is added to make it easier for the semantic segmentation task to focus on the image object. The complete convolution structure is adopted by the semantic segmentation branch, which ensures the overall running speed of the model. The loss function of the semantic segmentation branch is defined as follows:

(1) $$L_{seg}\left(X,C;\theta_{seg}\right)=-\frac{1}{\left|B\right|\cdot H\cdot W}W_{seg}\sum_{k\in \left|C\right|}\sum_{i\in P}\log\left[Soft\max\left(a_{k}^{i}\right)\right],$$

where |B| is the batch size of an iterative image, H and W are the number of pixels of the image X height and width respectively, P is the set of pixels in the whole image, $a_{k}^{i}$ denotes the mark score of the i pixel in the image for category k by the current model. C denotes the total number of categories under the task. $\theta_{seg}$ denotes the parameters of the semantic segmentation branch. $W_{seg}$ denotes the weight and offset of the semantic segmentation branch.

### 2.4. Prediction Part

The multi-scale features are used to predict the location and category of the image object. The main idea is that each layer of features generated by the hourglass structure is individually predicted, and the features are input into two 3×3 convolutional layers with different numbers of convolution kernels. The two generated feature maps are one for classification and one for the location of the regression detection box. Finally, the non-maximum suppression (NMS) [10,33] algorithm is used to remove the redundant region proposal.

The loss function is composed of positioning error ($L_{loc}\left(x,l,g\right)$) and classification error ($L_{conf}\left(x,c\right)$). The loss function is the average value of the weighted sum of the classification and regression errors of each region proposal. The average value is used to ensure that the gradient is not too large in the training process. The loss function of the prediction part is defined as follows:

(2) $$L\left(x,c,l,g;\theta_{pre}\right)=\frac{1}{N}W_{pre}\left(L_{conf}\left(x,c;\theta_{pre}\right)+\alpha L_{loc}\left(x,l,g;\theta_{pre}\right)\right),$$

where *N* is the number of matched detection boxes, α is a settable parameter, and α is set to 1. $\theta_{pre}$ denotes the parameters of the prediction part. $W_{pre}$ denotes the weight and offset of the prediction part.

The positioning error is smooth L1 loss [12], which is used to describe the positioning gap between the detection box ($l=\left(l^{x},l^{y},l^{w},l^{h}\right)$) and the callout box ($g=\left(g^{x},g^{y},g^{w},g^{h}\right)$). These boxes are represented by a four-tuple, which are the *x* and *y* coordinates of the center point of the box, and the width and height of the box. The positioning error is defined as follows:

(3) $$L_{loc}\left(x,l,g;\theta_{pre}\right)=\sum_{i,j,u\in \{x,y,w,h\}}^{}x_{ij}^{k}smooth_{L1}\left(l_{i}^{u}-g_{j}^{u}\right),$$

(4) $$smooth_{L1}\left(x\right)=\left\{ \begin{array}{ll} 0.5x^{2} & if\;\left|x\right|<1\\ \left|x\right|-0.5 & otherwise \end{array}\right.,$$

where $x_{ij}^{k}$ denotes whether the algorithm detection frame i and the real frame j match for category k. If it matches, the value of $x_{ij}^{k}$ is 1, else the value of $x_{ij}^{k}$ is 0. 

The classification loss is defined as follows:

(5) $$L_{conf}\left(x,c;\theta_{pre}\right)=-\sum_{i,j}^{}x_{ij}^{k}\log(\hat{c_{i}^{k}})-\sum_{i}\log\left(\hat{c_{i}^{k}}\right),$$

where $x_{ij}^{k}$ denotes whether the algorithm detection box i and the real box j match for the category k, $\hat{c_{i}^{k}}$ denotes the predicted probability of the *i*-th detection box corresponding to category k, which is expressed in the form of SoftMax [34]. 

The loss function is the addition of the loss function of the prediction module and the semantic segmentation branch, which is defined as follows:

(6) $$L_{loss}=L\left(x,c,l,g;\theta_{pre}\right)+L_{seg}\left(X,C;\theta_{seg}\right).$$

### 2.5. Summary of the Proposed Algorithm

Our method is summarized in Algorithm 1. In the training stage, first, the images are batch-input into the model. Second, the gradient function is used to minimize the loss function to train the neural network. Finally, the model with the highest object detection accuracy is selected according to the validation set. Note that both [5] and this paper are one-stage learning methods; however, the emphasis of each approach is entirely different. First, the shallow features are used in [5] to predict the object, but the hourglass structure is used by our method to integrate deep features and shallow features. Second, the method in [5] cannot fully capture more detailed information of the image, but our method can obtain more semantic information using the semantic segmentation task and attention mechanism.
**Algorithm 1** Entire process of the proposed algorithm 1:**Initialization:**Parameters $W_{seg}$ of the semantic segmentation branch;

Parameters $W_{pre}$ of the prediction part;

mini-batch size batch_size = 32. 2:**update until convergence:** 3:  **for** k step **do** 4:    Randomly sample batch_size points to construct a mini-batch. 5:    For each sampled point in the mini-batch, calculate Equations (1), (2), and (5) using forward propagation. 6:    Calculate the derivatives $\nabla_{W_{seg}}$ and $\nabla_{W_{pre}}$ by Adam algorithm [35]. 7:    Update the parameters $W_{seg}$ and $W_{pre}$ using backpropagation. 8:  **end for**


## 3. Experiment

### 3.1. Experimental Setting

In our experiments, we used the Intel Xeon E5-2603 v2 (dual CPU), NVIDIA 1080Ti GPU, 32GB memory, and Ubuntu x64 operating system. We selected the open source TensorFlow framework, Python 3.5 as the development environment and PyCharm2017.1.2 as the development tool.

We used two well-labeled benchmark datasets for object detection: Pascal VOC 2012 dataset [36] and Microsoft COCO (MS COCO) dataset [37], which came from image sensors. Pascal VOC 2012 dataset consists of 11,540 images of 20 categories. We randomly selected 5717 images as the training set and 5823 images as the test set. MS COCO dataset involves 91 categories, but only 80 categories are used in detection tasks. We also perform experiments on the 80 categories MS COCO dataset marked by Microsoft. We train using the 80k train images, and test on the 40k test images.

To verify the effectiveness of the algorithm for images, the algorithm in this paper is compared with SSD [19], Faster R-CNN [13], and Mask R-CNN [14]. SSD is a one-stage detection algorithm. Faster R-CNN and Mask R-CNN are two-stage detection algorithms.

IOU (intersection over union) [36] is used to evaluate the positioning accuracy effect. In object detection tasks using images, common evaluation indices include average precision (AP) [14] and mean average precision (mAP) [38]. AP represents the average precision, which is the average value of the highest precision rate under different recall rates. mAP is the mean value of AP under all categories. By default, in this paper, when the IOU between the detection box and the real box is greater than 0.5, it is determined that the algorithm has located the object.

We report the standard MS COCO indices [14,39], including AP (averaged over IOU thresholds), ${\mathrm{AP}}^{\mathrm{IoU}=0.50}$, ${\mathrm{AP}}^{\mathrm{IoU}=0.75}$ and ${\mathrm{AP}}^{\mathrm{small}}$, ${\mathrm{AP}}^{\mathrm{medium}}$, ${\mathrm{AP}}^{\mathrm{large}}$.

For the same test video stream, the number of frames per second processed under the same experimental conditions is used as the detection speed evaluation standard, which is abbreviated as fps [40]. It includes the image reading process and object detection process. We use the detection speed to evaluate the processing efficiency of the real-time detection algorithm for images.

### 3.2. Comparison of State-of-the-Art Algorithms

Table 2 shows the mAP values of the proposed method and other three-comparison methods on the Pascal VOC 2012 dataset. Comparing Mask R-CNN with the proposed method, the average mAP value of the Pascal VOC 2012 dataset increased from 74.9 to 79.4%. By analyzing the detection accuracy of each category in the dataset, it can be found that the detection accuracy of objects on the street have been greatly improved.

**Figure 4:**
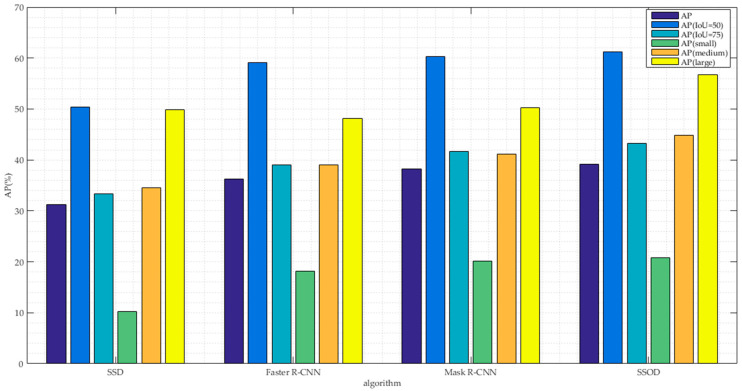
Comparison of our method and other state-of-the-art methods in terms of AP values (%) on the MS COCO dataset.

Figure 4 shows the mAP values of the proposed method and the other three methods on the MS COCO dataset. In the experiment, the proposed method showed extremely strong detection performance, which is better than the best performance of Mask R-CNN. In addition, the AP value of the large object is improved higher, and the model is more suitable for detecting the large object like the traditional one-stage algorithm. Experimental results show that this algorithm can also get good detection accuracy for complex environment of image.

### 3.3. Speed and Performance

Table 3 shows the mAP values (%) and detection speed (fps) of the proposed algorithm and the three comparison algorithms in terms of the Pascal VOC 2012 dataset. The detection speed of the SSOD algorithm is higher than that of the Faster R-CNN algorithm and Mask R-CNN algorithm, but the detection speed is lower than the SSD algorithm due to the complex model structure. The SSOD algorithm can achieve real-time detection speed, and the mAP of the algorithm is the highest among the comparison algorithms, which can be used for real-time detection of image sensors.

### 3.4. Further Analysis

#### 3.4.1. Comparison of the Variant Algorithms

We evaluate some variants of SSOD, i.e., SSOD-att-seg, SSOD-att. SSOD-att is based on semantic segmentation without attention mechanism. SSOD-att-seg represents a fast object detection algorithm based on hourglass network without semantic segmentation and attention mechanism. For fair comparison, the two variants remain the same configuration and hyperparameters as SSOD.

Table 4 shows the mAP results of the SSOD algorithm and other two variants of the SSOD algorithm. The best accuracy is shown in boldface. We can find that SSOD can achieve higher accuracy than SSOD-att and SSOD-att-seg. Compared with the SSOD-att algorithm, the mAP value of the SSOD algorithm is improved by 0.7%, and compared with the SSOD-att-seg algorithm by 3%. However, the overall detection accuracy of the SSOD-att-seg algorithm is higher than the three comparison algorithms (SSD, Faster R-CNN, and Mask R-CNN).

**Figure 5:**
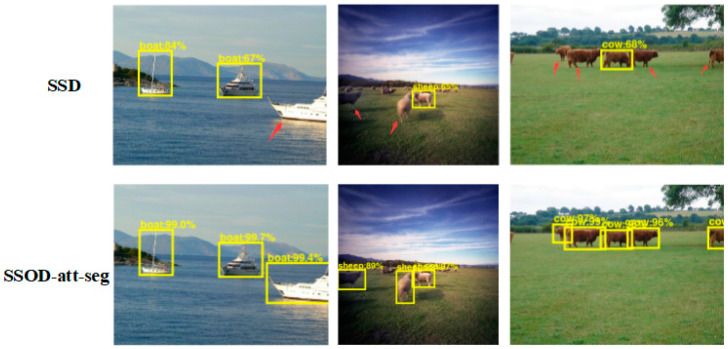
Comparison of detection effects between the SSOD-att-seg algorithm and SSD algorithm. The arrows indicate the objects detected by the SSOD-att-seg algorithm but missed by the SSD algorithm.

By analyzing the detection accuracy of each type of object in the SSOD-att-seg algorithm, it can be found that the detection accuracy of SSOD-att-seg for the object with large positions in the picture, such as boats, sofas, and trains, is greatly improved, indicating that the SSOD-att-seg algorithm can better detect the big object in the picture. Compared with Mask R-CNN, SSOD-att-seg has no advantage in recognition accuracy of some small objects, because the one-stage detection model has a disadvantage in detecting small objects compared with the two-stage model. However, the SSOD-att-seg algorithm has an improved average accuracy compared with the SSD which is the same one-stage algorithm. Figure 5 shows the comparison of the detection effect between the SSOD-att-seg algorithm and SSD algorithm. In the figure, the detection confidence of SSOD-att-seg is higher and there are fewer undetected objects, which indicates that the idea of SSOD-att-seg using the hourglass structure to generate multi-scale features can effectively improve the detection accuracy of small objects in images.

**Figure 6:**
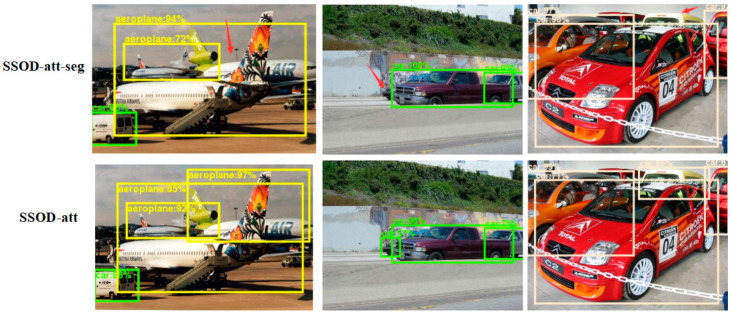
Comparison of detection effect between the SSOD-att-seg algorithm and SSOD-att algorithm. The arrows indicate the objects detected by the SSOD-att algorithm but missed by the SSOD-att-seg algorithm.

By analyzing the detection accuracy of each category of the SSOD-att algorithm, it can be found that the detection accuracy of the SSOD-att algorithm is greatly improved for boats, potted plants, aeroplanes, and other objects that are easy to be partially blocked in pictures. Figure 6 shows the comparison of the detection results of the SSOD-att algorithm and SSOD-att-seg algorithm.

The detection confidence of SSOD-att is higher and there are fewer undetected objects. Experiments have proved that the improved model using the multi-task learning idea can better detect the partially occluded objects in the images, and can be used for detection in some scenes with dense objects.

**Figure 7:**
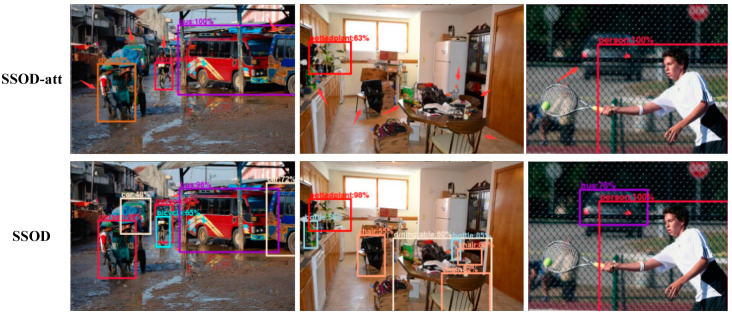
Comparison chart of detection effect between the SSOD algorithm and SSOD-att algorithm. The arrows indicate the objects detected by the SSOD algorithm but missed by the SSOD-att algorithm.

By observing the pictures detected by the algorithm, it is also found that the SSOD-att algorithm is not accurate enough to detect objects, which is caused by the complex background at home, and the complex background may cause the false detection of the algorithm. Figure 7 shows the comparison of the detection results of the SSOD algorithm and the SSOD-att algorithm. The detection confidence of SSOD is higher and there are fewer undetected objects. The experiments proved that the SSOD algorithm can well alleviate the false detection problem caused by the interference of the cluttered background in the images.

#### 3.4.2. Speed and Performance

Table 5 shows the average accuracy and speed of the SSOD-att-seg algorithm, SSOD-att algorithm, and SSOD algorithm on the Pascal VOC 2012 dataset. The detection speed of the SSOD-att-seg algorithm, SSOD-att algorithm, and SSOD algorithm is higher than the Faster R-CNN algorithm and Mask R-CNN algorithm. However, due to the complex model structure, the detection speed of the SSOD-att-seg algorithm, SSOD-att algorithm, and SSOD algorithm is lower than that of the SSD algorithm. SSOD-att-seg, SSOD-att, and SSOD algorithms can achieve real-time in detection speed. The SSOD algorithm is the highest average accuracy rate among the three comparison algorithms, and can be used for real-time detection of image sensors.

## 4. Conclusions

In this paper, we proposed an object detection algorithm combined with semantic segmentation for images. The algorithm in this paper uses the hourglass structure to regenerate multi-scale features, and the feature mapping used to predict small targets also has rich abstract features. Semantic segmentation is introduced as an auxiliary task of object detection. Semantic segmentation and target detection are performed in parallel to optimize the model together. Extensive experimental results show that the performance of the object detection algorithm in this paper has been significantly improved, and the detection speed can reach real-time. Comparing state-of-the-art Mask R-CNN with the SSOD method, the average mAP value increased by 4.5% on the Pascal VOC 2012 dataset. Comparing state-of-the-art Mask R-CNN with the SSOD method, the 5 AP indices value has significant improvement on the MS COCO dataset, especially helpful for medium and large objects, improving APs by 3.7% and 6.5% respectively. The detection speed of SSOD is 24 frames per second, which can be used for real-time detection. The training time of the proposed method is about 22 h. Note that data-parallel training algorithms cannot be considered for images in our paper, which will be our future work.

## Figures and Tables

**Table 1:** Detailed parameters of hourglass structure.

Network Layer	Kernel Parameter	Repeat	Output
Input	10×10×2048		
Block1	$\left[\begin{array}{l} 1\times 1,1024\\ 3\times 3,1024\\ 1\times 1,1024 \end{array}\right]$	2	5×5×1024
Block2	$\left[\begin{array}{l} 1\times 1,1024\\ 3\times 3,1024\\ 1\times 1,1024 \end{array}\right]$	4	3×3×1024
Block3	$\left[\begin{array}{l} 1\times 1,512\\ 3\times 3,512\\ 1\times 1,512 \end{array}\right]$	1	3×3×512
Block4	$\left[\begin{array}{l} 1\times 1,512\\ 3\times 3,512\\ 1\times 1,512 \end{array}\right]$	1	5×5×512
Block5	$\left[\begin{array}{l} 1\times 1,512\\ 3\times 3,512\\ 1\times 1,512 \end{array}\right]$	1	10×10×512
Block6	$\left[\begin{array}{l} 1\times 1,512\\ 3\times 3,512\\ 1\times 1,512 \end{array}\right]$	1	19×19×512
Block7	$\left[\begin{array}{l} 1\times 1,512\\ 3\times 3,512\\ 1\times 1,512 \end{array}\right]$	1	38×38×512

**Table 2:** Comparison of our method and other state-of-the-art methods in terms of mAP values (%) on the Pascal VOC 2012 dataset. The best accuracy is shown in boldface.

Number	Category	Faster R-CNN (%)	SSD (%)	Mask R-CNN (%)	SSOD (ours) (%)
1	aeroplane	84.9	85.6	**86.5**	84.3
2	bicycle	79.8	80.1	83.9	**87.8**
3	bird	74.3	70.5	77.6	**79.4**
4	boat	53.9	57.6	58.8	**73.2**
5	bottle	49.8	46.2	**63.7**	54.5
6	bus	77.5	79.4	80.4	**84.3**
7	car	75.9	76.1	78.2	**86.1**
8	cat	88.5	**89.2**	87.6	87.1
9	chair	45.6	53.0	56.0	**60.8**
10	cow	77.1	77.0	80.1	**85.0**
11	dining table	55.3	60.8	63.2	**80.2**
12	dog	86.9	87.0	**88.0**	86.6
13	horse	81.7	83.1	84.4	**88.1**
14	motorbike	80.9	82.3	**85.7**	84.5
15	person	79.6	79.4	**79.9**	79.8
16	potted plant	40.1	45.9	46.3	**56.2**
17	sheep	72.6	75.9	74.4	**81.1**
18	sofa	60.9	69.5	66.8	**80.8**
19	train	81.2	81.9	81.3	**87.5**
20	tv/monitor	61.5	67.5	75.0	**80.6**
-	mAP	70.4	72.4	74.9	**79.4**

**Table 3:** Comparison of our algorithm and other state-of-the-art algorithms in terms of mAP values (%) and detection speed (fps) on the Pascal VOC 2012 dataset. The best accuracy or detection speed is shown in boldface.

Algorithm	SSD	Faster R-CNN	Mask R-CNN	SSOD
mAP (%)	72.4	70.4	74.9	**79.4**
detection speed (fps)	**46**	5	5	24

**Table 4:** Comparison of our algorithm and two variants in terms of mAP values (%) on the Pascal VOC 2012 dataset. The best accuracy is shown in boldface.

Number	Category	SSOD-att-seg (%)	SSOD-att (%)	SSOD (%)
1	aeroplane	88.4	**90.1**	84.3
2	bicycle	83.1	82.7	**87.8**
3	bird	75.5	**85.2**	79.4
4	boat	62.5	**74.0**	73.2
5	bottle	46.9	48.3	54.5
6	bus	82.5	**90.4**	84.3
7	car	79.3	75.2	**86.1**
8	cat	**92.4**	87.9	87.1
9	chair	59.2	51.5	**60.8**
10	cow	81.3	81.4	**85.0**
11	dining table	65.9	75.8	**80.2**
12	dog	**90.1**	87.3	86.6
13	horse	84.5	82.8	**88.1**
14	motorbike	86.8	**92.0**	84.5
15	person	82.5	**84.2**	79.8
16	potted plant	51.5	**58.0**	56.2
17	sheep	80.9	**83.2**	81.1
18	sofa	75.7	71.1	**80.8**
19	train	85.8	**88.2**	87.5
20	tv/monitor	72.1	**84.9**	80.6
-	mAP	76.4	78.7	**79.4**

**Table 5:** Comparison of our algorithm and two variants in terms of mAP values (%) and detection speed (fps) on the Pascal VOC 2012 dataset. The best accuracy or detection speed is shown in boldface.

Algorithm	SSOD-att-seg	SSOD-att	SSOD
mAP (%)	76.4	78.7	**79.4**
detection speed (fps)	**25**	24	24
